# Oncogenic microRNA-411 promotes lung carcinogenesis by directly targeting suppressor genes SPRY4 and TXNIP

**DOI:** 10.1038/s41388-018-0534-3

**Published:** 2018-11-02

**Authors:** Caiyan Zhang, Huimin Wang, Xiaomin Liu, Yanping Hu, Lei Ding, Xing Zhang, Qiangling Sun, Yanli Li

**Affiliations:** 1 Lab for Noncoding RNA and Cancer, School of Life Sciences, Shanghai University, Shanghai, 200444 China; 2 School of Environmental Science and Engineering, Shanghai University, Shanghai, 200444 China; 3 School of Life Science and Technology, China Pharmaceutical University, Nanjing, 210009 China; 4 Central Laboratory, Shanghai Chest Hospital, Shanghai Jiaotong University, Shanghai, 200030 China

**Keywords:** Growth factor signalling, Non-small-cell lung cancer

## Abstract

Lung cancer is one of the most common malignant diseases globally, composed of non-small cell lung cancer (NSCLC, 85%) and small cell lung cancer (SCLC, 15%). MicroRNAs (miRNAs) are single-stranded noncoding RNAs having important roles in lung cancer development. miR-411-5p/3p were reported to be increased significantly in human NSCLC tissues and cell lines. Moreover, miR-411-5p/3p overexpression could accelerate cell proliferation and migration, and impede cell apoptosis in NSCLC cell lines. Mechanically, SPRY4 is confirmed a direct target of miR-411-5p/3p. Furthermore, our findings showed that miR-411-5p/3p promoted lung tumor growth in vivo, decreased SPRY4 expression dramatically, and induced EGFR, AKT signaling activation, as well as epithelial–mesenchymal transition (EMT) simultaneously in tumor tissues. In addition, we showed that miR-411-5p also targeted tumor suppressor TXNIP, involved in regulating positively cell cycle progress in SPC-A1 cells rather than in H1299. Whether cell specificity of low TXNIP mRNA level in H1299 is responsible for the different response to cell cycle between H1299 and SPC-A1 would need further explorations. Collectively, these results suggest that miR-411-5p/3p are required for NSCLC development by suppressing SPRY4 and TXNIP; thus, the miR-411-SPRY4-AKT axis might act as a promising target for lung cancer therapy clinically.

## Introduction

Lung cancer is the leading cause of death globally and is responsible for higher morbidity among cancers [1]. Non-small cell lung cancer (NSCLC) occupies around 85% of lung cancer. As diagnoses are often made when the disease is advanced or metastatic, the 5-year survival rate is ~15% [2]. MicroRNAs (miRNAs) are single-stranded small noncoding RNAs with a length of 18–25 nucleotides (nt), which exist widely in eukaryotic organisms [3] with nearly 60% of genes under their regulation posttranscriptionally mainly via direct interacting the 3′-untranslated region (3′-UTR) of mRNAs, leading to either mRNA degradation or translational repression [4, 5].

In tumor processes, miRNAs have critical roles in cell proliferation [6], apoptosis [7], migration [8], stem cell differentiation [9], and the production of cancer stem cell [10, 11]. Such information suggests that miRNAs are involved in tumorigenesis and can be potential targets for drug development.

Sprouty (SPRY) are inhibitors of receptor tyrosine kinase modulating tracheal branching in *Drosophila* and their alternative splicing results in multiple transcript variants (SPRY1, SPRY2, SPRY3, and SPRY4) [12–14], which are reported to downregulate the expression of epidermal growth factor receptor (EGFR) [15]. *SPRY4* functions as a tumor suppressor downstream of Wnt7A/Fzd9 signaling in lung cancer, whose overexpression inhibited cell growth with upregulating the tumor suppressor p53 and p21 expression, and also suppressed cell migration and invasion along with MMP-9 activity [16]. *SPRY4* is activated by a target downstream of Wnt7A/Fzd9 signaling [17], PPARγ, which has vital roles in ovarian cancer [18], colorectal cancer [19], and prostate cancer [20], and affects cell growth, differentiation, and metastasis [16]. In melanoma [21], breast cancer [22], and prostate cancer [23], SPRY4 inhibits cell migration and the cancer stem cell properties of breast carcinoma cells [24]. Nevertheless, as an oncogene, SPRY4 promotes ovarian cancer invasion through involvement in EGFR-mediated human ovarian cancer progression [25]. Thioredoxin interaction protein (TXNIP) has pivotal roles in prostate cancer, lung cancer, and breast cancer [26–28], and has an especially prognostic effect in NSCLC [29].

MiR-411 belongs to the 14q32.31 miRNA cluster [30]. In the present study, we confirmed SPRY4 as a common target of miR-411-5p and miR-411-3p. Moreover, miR-411-5p/3p could promote NSCLC cell proliferation, tumor growth, and metastasis in vitro and in vivo. These results indicated that the miR-411 could be a cancer driver in lung tumorigenesis.

## Results

### MiR-411 is upregulated in human NSCLC tissues and cell lines

**Fig. 1:**
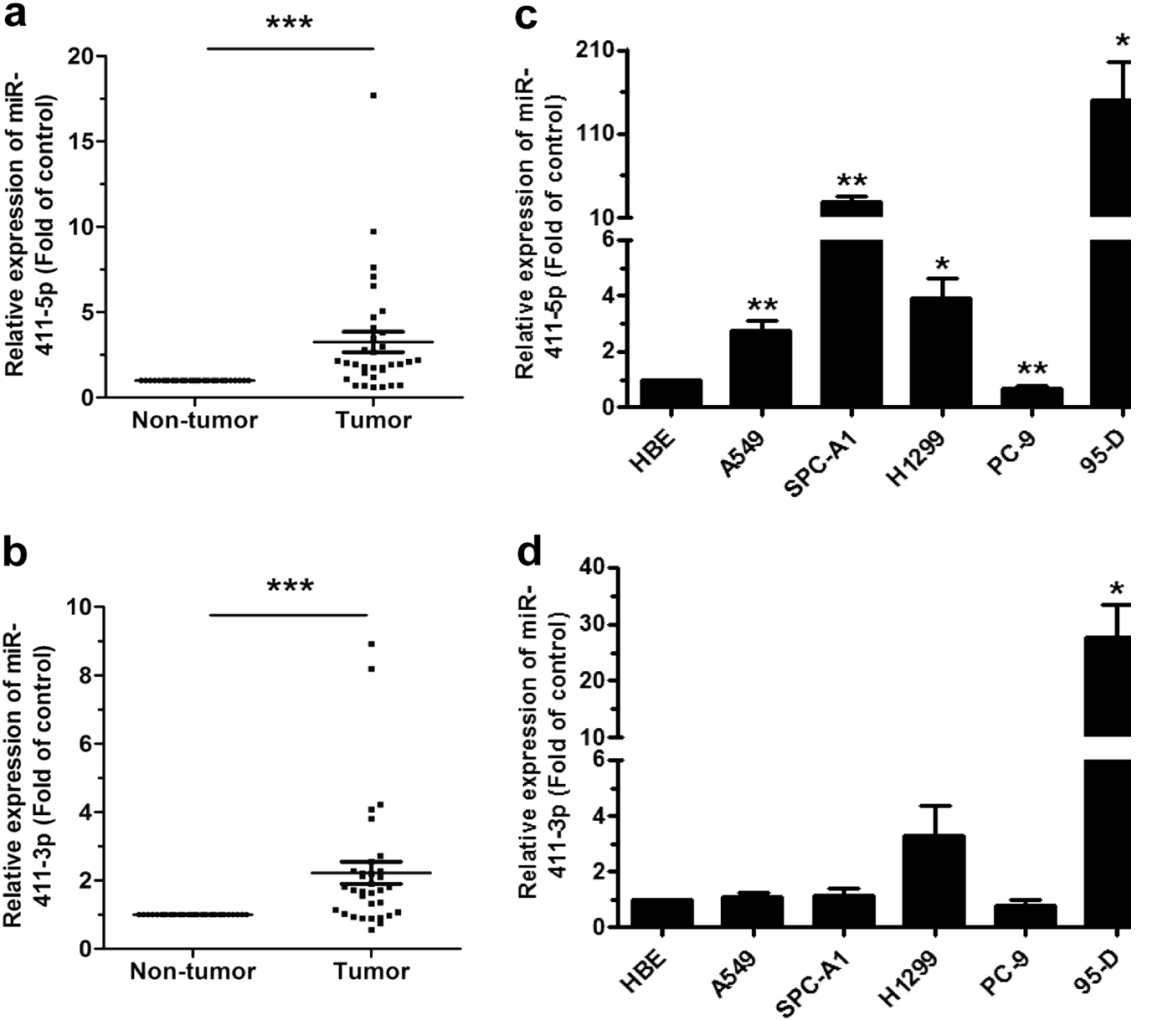
MiR-411 expression was upregulated in NSCLC. **a**, **b** Relative miR-411-5p/3p expression in NSCLC and corresponding paracancerous lung tissues (*n* *=* 33). **c**, **d** MiR-411-5p/3p expression in NSCLC cell lines A549, SPC-A1, H1299, PC-9, and 95-D. HBE cells was the normal control. *P *<* 0.05, **P *<* 0.01, ***P *<* 0.001

We investigated the miR-411-5p/3p expression in human NSCLC tissue samples and cell lines. Results of quantitative reverse-transcriptase PCR (qRT-PCR) indicated that the miR-411-5p/3p expression was significantly higher in the 33 human lung tumor samples than in those of adjacent non-tumor tissues (Fig. 1a, b). It was also observed that miR-411-5p/3p were upregulated in most human NSCLC cell lines compared with the normal bronchial epithelium cell line HBE135-E6E7 (HBE, Fig. 1c, d). The results indicated that miR-411 could function as an oncogene.

### MiR-411-5p/3p promote cell proliferation and inhibit cell apoptosis in NSCLC cell lines

**Fig. 2:**
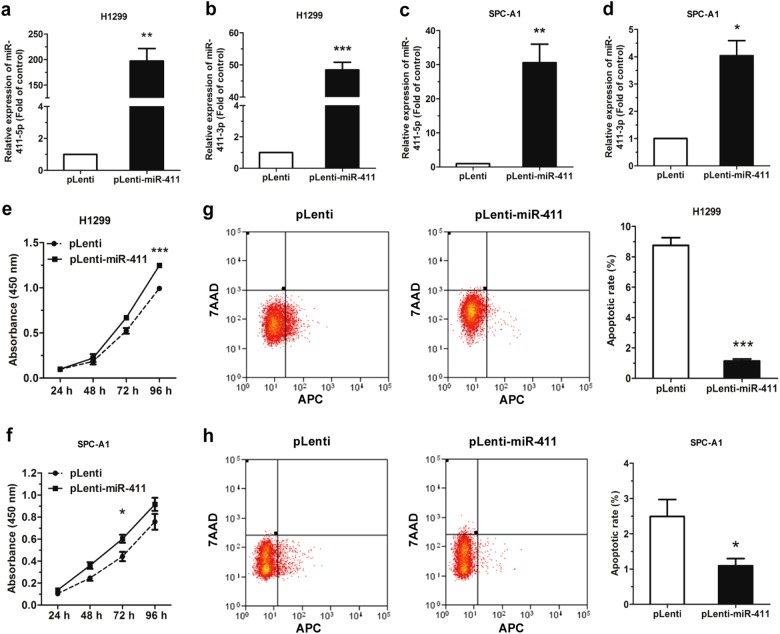
miR-411-5p/3p promote cell proliferation and impeded apoptosis in NSCLC cell lines. **a**–**d** miR-411-5p/3p expression was upregulated in pLenti-miR-411 H1299 and SPC-A1 cells compared to pLenti cells. **e**, **f** Cell proliferation of pLenti/pLenti-miR-411 H1299 and SPC-A1 was assessed by CCK-8 assay. **g**, **h** Flow cytometry was used to analyze the apoptosis of pLenti/pLenti-miR-411 H1299 and SPC-A1 cells. *P *<* 0.05, **P *<* 0.01, ***P *<* 0.001

We used H1299 and SPC-A1 cells stably transfected with miR-411-5p/3p (pLenti-miR-411 H1299/SPC-A1) or an empty vector (pLenti H1299/SPC-A1) for assessment of the effect of miR-411-5p/3p in NSCLC. The green fluorescence protein-positive pLenti and pLenti-miR-411 H1299 cells were nearly 99% (Supplementary Figure S1). The miR-411-5p expression was increased significantly in the pLenti-miR-411 H1299 and SPC-A1 cells compared with the pLenti cells, with approximately 200- and 30-folds of increase, respectively (Fig. 2a–c), and miR-411-3p expression was upregulated by ~48- and 4-folds of increase, respectively (Fig. 2b–d).

Results from the CCK-8 assay showed that miR-411-5p/3p overexpression dramatically promoted cell proliferation of H1299 and SPC-A1 cells (Fig. 2e, f). Furthermore, we revealed that pLenti-miR-411 promoted cell cycle progression significantly in SPC-A1 cells compared with the pLenti cells (Fig. S2a). However, no significant difference was observed on cell cycle progression in H1299 cells, which might result from the variable mRNA expression level of TXNIP in different cells (Fig. S2b).

We then investigated the impact of miR-411-5p/3p on cell apoptosis through staining with Annexin-V Allophycocyanin (APC) and 7-aminoactinomycin D (7AAD) followed by flow cytometry. Results showed the apoptotic cell rate was remarkably reduced in the pLenti-miR-411 H1299 and SPC-A1 cells in comparison with the pLenti cells (Fig. 2g, h).

### MiR-411-5p/3p induce cell migration in NSCLC cell lines

**Fig. 3:**
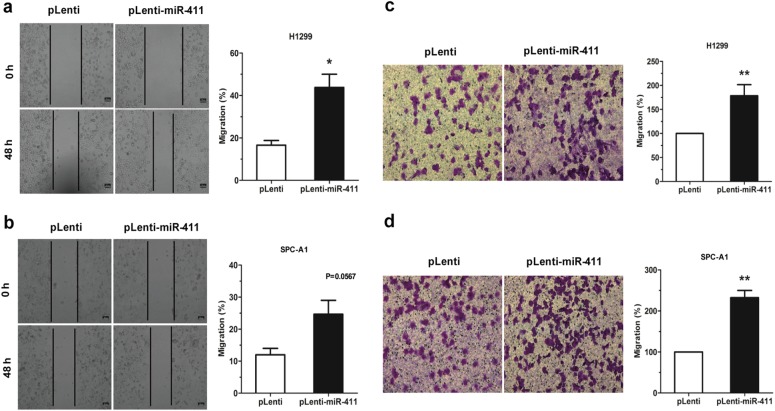
MiR-411 induce cell migration in NSCLC cell lines. **a**, **b** Wound-healing assay of pLenti/pLenti-miR-411 H1299 and SPC-A1 cells. The relative position of cells was recorded at 0 h and 48 h, respectively. **c**, **d** Transwell assay of pLenti/pLenti-miR-411 H1299 and SPC-A1 cells. The migrated cells were fixed, stained, and photographed 48 h post incubation and then the cell numbers were counted. *P *<* 0.05, **P *<* 0.01

To assess the impact of miR-411-5p/3p on cell motility, wound-healing assay was performed using the pLenti-miR-411 H1299 and SPC-A1 cells, and photographed the lesions at 0 and 48 h to record the movement of cells. In the pLenti-miR-411 H1299 and SPC-A1 cells, cell motility was reduced in comparison with pLenti cells (Fig. 3a, b). The same results were recapitulated in a transwell assay (Fig. 3c, d).

### Inhibition of miR-411-5p/3p promotes apoptosis and suppresses cell migration in NSCLC cell lines

**Fig. 4:**
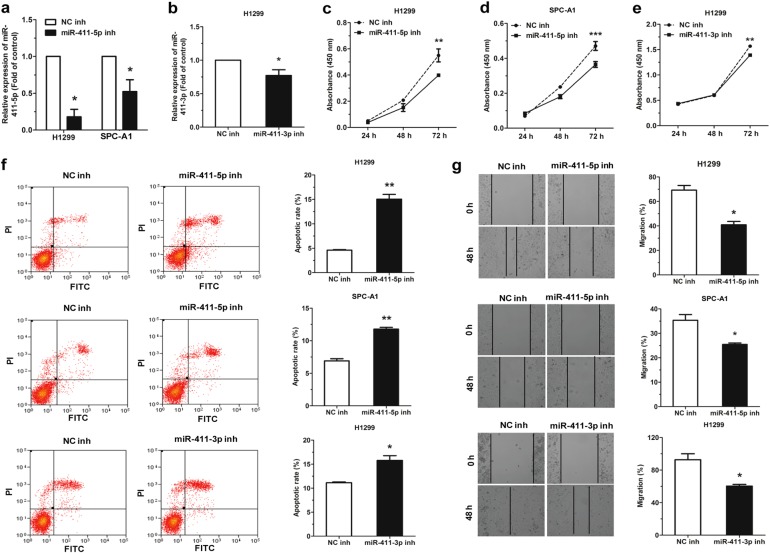
miR-411-5p/3p inhibition induces apoptosis and suppressed migration in NSCLC cell lines. **a** Relative expression of miR-411-5p in H1299 and SPC-A1 cells with transfection of NC inhibitor (NC inh) and miR-411-5p inhibitor (miR-411-5p inh). **b** Relative expression of miR-411-3p in H1299 cells with transfection of NC inh miR-411-3p inhibitor (miR-411-3p inh). **c**–**g** The effect of miR-411-5p/3p inhibition on proliferation (**c**–**e**), apoptosis (**f**), and migration (**g**) in H1299 and SPC-A1 cells. *P *<* 0.05, **P *<* 0.01, ***P *<* 0.001

For further elucidation of miR-411 functions in NSCLC cells, H1299 and SPC-A1 cells were transfected with negative control inhibitor (NC inh) and miR-411-5p or miR-411-3p inhibitor (miR-411-5p inh or miR-411-3p inh).The expression of miR-411-5p achieved ~80% and 50% decreases in the two cell lines, respectively (Fig. 4a), and that of miR-411-3p declined about 20% in H1299 cells (Fig. 4b).

It was then observed that the miR-411-5p/3p inhibition impeded the cell proliferation capacity of the H1299 and SPC-A1 cells (Fig. 4c–e), and promoted cell apoptosis (Fig. 4f). Reduction of miR-411-5p/3p expression also reduced cell migration (Fig. 4g).

### MiR-411-5p/3p directly target SPRY4

**Fig. 5:**
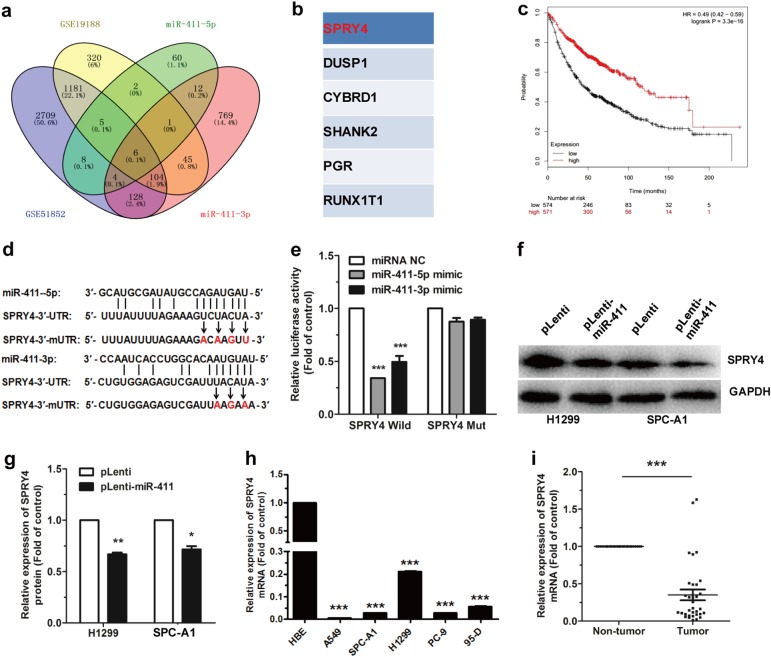
miR-411-5p/3p directly target SPRY4. **a** Six candidate genes were obtained by miRWalk 2.0 plus two sets of microarray data (GSE51852 and GSE19188). **b** The six candidate genes, SPRY4, DUSP1, CYBRD1, SHANK2, PGR, and RUNX1T1. **c** SPRY4 was associated with longer overall patient survival. **d** The seed regions of miR-411-5p/3p of SPRY4 3′-UTR and 3′-mUTR. **e** The luciferase activity of HEK293T cells co-transfected with NC mimic or miR-411-5p/3p mimic, and pGL3-SPRY4-3′-UTR (SPRY4 Wild) or pGL3-SPRY4-3′-mUTR (SPRY4 Mut). **f**–**h** SPRY4 protein expression (**f**, **g**) and mRNA expression (**h**) were determined by western blotting and qRT-PCR, respectively, in pLenti/pLenti-miR-411 H1299 and SPC-A1 cells. **i** SPRY4 mRNA expression was measured by qRT-PCR in patient tissues. *P < 0.05, **P < 0.01, ***P < 0.001

To explore the mechanism of miR-411-5p/3p promoting NSCLC progress at the molecule level, miRWalk 2.0 is used for potential target genes prediction in combination with two sets of lung cancer microarray data (GSE51852 and GSE19188). Six candidate genes in total were obtained. The candidate genes were selected by which expression down to 0.5 times in GSE51852 and GSE19188 (Fig. 5a, b).

SPRY4 expression was associated with longer overall patient survival (Fig. 5c) (http://kmplot.com/analysis/index.php?p=service&start=1). The binding sites for miR-411-5p/3p with SPRY4 wild-type 3′-UTR (SPRY4 3′-UTR) and SPRY4 mutant 3′-UTR (SPRY4 3′-mUTR) were shown (Fig. 5d). Dual luciferase reporter assay was used to demonstrate a significant luciferase activity decrease ( > 50%) in HEK293T cells with co-transfection of pGL3-SPRY4 wild-type 3′-UTR (pGL3-SPRY4-3′-UTR), pRL vector, and miR-411-5p/3p mimic compared with the control (co-transfection of pGL3-SPRY4-3′-UTR, pRL vector, and NC mimic). As contrast, co-transfection of miR-411-5p/3p with the pGL3-SPRY4 mutant 3′-UTR (pGL3-SPRY4-3′-mUTR) showed no remarkable change in luciferase activity, indicating the direct binding between miR-411-5p/3p and the SPRY4 3′-UTR (Fig. 5e).

To assess whether or not the expression of SPRY4 has been affected in pLenti-miR-411 H1299 and SPC-A1 cells in comparison with that in pLenti cells, we investigated the SPRY4 protein expression. Results revealed that miR-411-5p/3p decreased SPRY4 expression on the protein levels in H1299 and SPC-A1 cells (Fig. 5f, g). Further results indicated that, SPRY4 was downregulated in human NSCLC cell lines as well as in lung cancer samples compared with the non-tumor tissues (Fig. 5h, i).

**Fig. 6:**
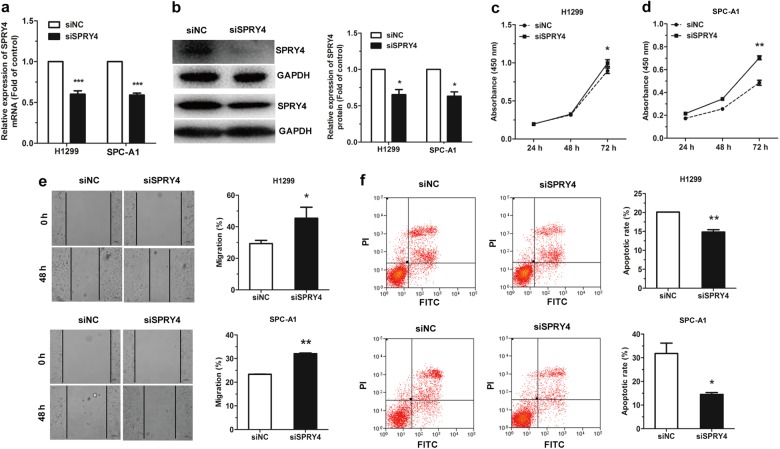
siSPRY4 effects on cell proliferation and migration in NSCLC. **a**, **b** siSPRY4 downregulated SPRY4 at mRNA (**a**) and protein (**b**) levels in H1299 and SPC-A1 cells, as determined by qRT-PCR and western blotting, respectively. **c**–**f** siSPRY4 effects on cell proliferation (**c**, **d**), migration (**e**), and apoptosis (**f**) in H1299 and SPC-A1 cells. *P < 0.05, **P < 0.01, ***P < 0.001

To investigate the effects of SPRY4 on NSCLC, SPRY4 small interfering RNA (siSPRY4) was transfected into H1299 and SPC-A1 cells. The expression of SPRY4 mRNA and protein was decreased markedly in both H1299 and SPC-A1 cells (Fig. 6a, b). Results showed significant acceleration on the proliferation and migration of cells with transfection of siSPRY4 (Fig. 6c–e). Meanwhile, the apoptotic cell rate was significantly decreased in those cells transfected with siSPRY4 (Fig. 6f). Collectively, SPRY4 silencing mimics the effects of miR-411-5p/3p overexpression in NSCLC cells.

### Overexpressing SPRY4 rescues the effects of miR-411

**Fig. 7:**
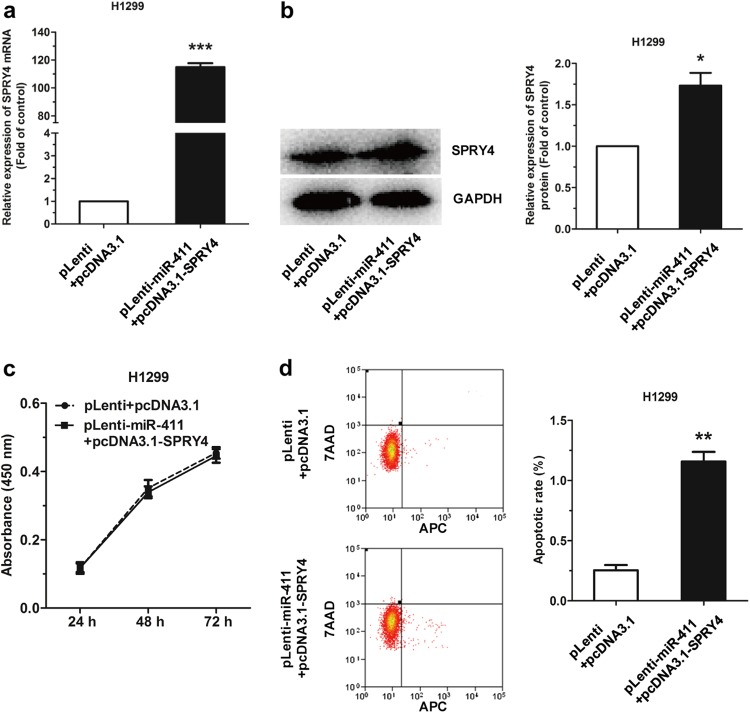
SPRY4 rescues the miR-411-5p/3p effects in H1299 cells. **a**, **b** SPRY4 mRNA and protein level in pLenti/pLenti-miR-411 H1299 cells transfected with pcDNA3.1 or pcDNA3.1-SPRY4. **c** Cell proliferation of pLenti-miR-411 H1299 with transfection of pcDNA3.1-SPRY4 was rescued compared with pLenti cells of pcDNA3.1. **d** Cell apoptosis rates of pLenti-miR-411 H1299 with transfection of pcDNA3.1-SPRY4 were analyzed by flow cytometry. The inhibition of miR-411 on H1299 apoptosis was rescued by pcDNA3.1-SPRY4. *P *<* 0.05, **P *<* 0.01, ***P *<* 0.001

To determine whether upregulation of SPRY4 expression was able to rescue cell proliferation and the reduction in cell apoptosis in the pLenti-miR-411 or pLenti H1299 cell line, a SPRY4 expression vector pcDNA3.1-SPRY4 was constructed. Overexpression of SPRY4 by pcDNA3.1-SPRY4 in H1299 cells inhibited cell proliferation and increased apoptosis with decreasing protein level of N-cadherin, EGFR, and p-AKT1, and increasing E-cadherin (Supplementary Fig. S3). pLenti-miR-411 H1299 cells or pLenti H1299 cells with pcDNA3.1-SPRY4 or pcDNA3.1 resulted in a marked upregulation of SPRY4 mRNA and protein expression by 110-folds and 1.8-folds, respectively (Fig. 7a, b). Results further revealed that pcDNA3.1-SPRY4 rescued the promotion effect on cell proliferation and the repressive effect on apoptosis in pLenti-miR-411 H1299 cells (Fig. 7c, d). These findings demonstrated that the effects of miR-411-5p/3p overexpression in NSCLC cells could be rescued by SPRY4 upregulation.

### TXNIP is a direct target of miR-411-5p

**Fig. 8:**
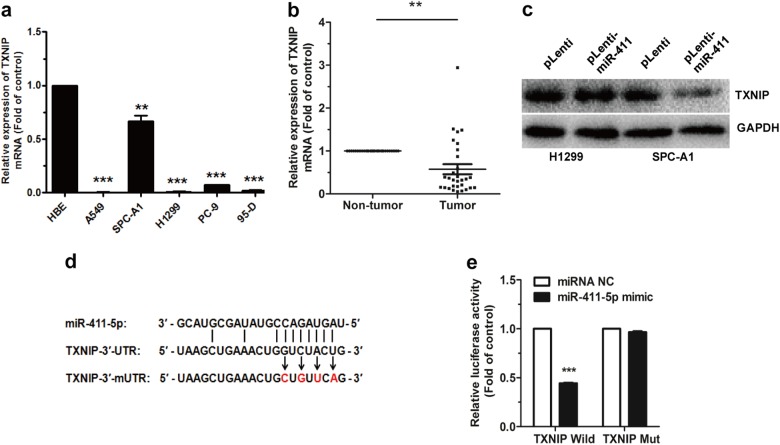
TXNIP is a direct target of miR-411-5p. **a** TXNIP mRNA expression in A549, SPC-A1, H1299, PC-9, and 95-D cells. HBE cell line was normal control. **b** TXNIP mRNA expression in NSCLC tissue samples and corresponding non-tumor tissues (*n* *=* 33). **c** TXNIP protein level was determined in pLenti/pLenti-miR-411 H1299 and SPC-A1 cells by western blotting. **d** The seed regions of miR-411-5p of TXNIP 3′-UTR and 3′-mUTR. **e** The luciferase activity of HEK293T cells co-transfected with NC mimic or miR-411-5p mimic and pGL3-TXNIP-3′-UTR (TXNIP Wild) or pGL3-TXNIP-3′-mUTR (TXNIP Mut). **P *<* 0.01, ***P *<* 0.001

As a suppressor gene in lung cancer, *TXNIP* is also confirmed to be a target of miR-411-5p and decreased in NSCLC cell lines and lung cancer tissue samples. (Fig. 8a, b). Next, we set out to assess the effect of repression of TXNIP in H1299 and SPC-A1 cells with pLenti-miR-411 compared with pLenti cells, and thus investigated the expression of TXNIP by western blotting, which was not surprisingly decreased in both H1299 and SPC-A1 cells. (Fig. 8c). It was further confirmed to be a direct target of miR-411-5p by dual luciferase reporter assay (Fig. .8d, e).

### MiR-411 is required for tumor growth in vivo

**Fig. 9:**
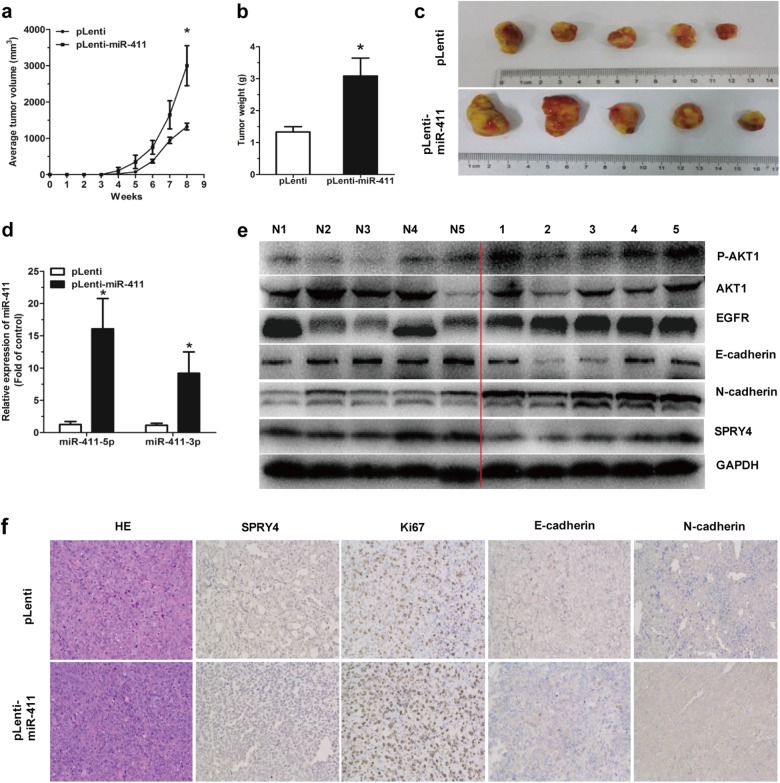
miR-411-5p/3p overexpression promotes tumor growth in vivo. **a** pLenti/pLenti-miR-411 H1299 cells were subcutaneously implanted into SCID mice with volumes of tumor recorded weekly. **b**, **c** Mice tumors were weighed (**b**) and photoed (**c**) 8 weeks post injection. **d** miR-411-5p/3p expression was measured in mice tumors by qRT-PCR. **e** SPRY4, E-cadherin, and N-cadherin protein levels were detected in mice tumors by western blotting. **f** HE staining (original magnifications × 200) of tumors and SPRY4, Ki67, E-cadherin, and N-cadherin protein levels were determined by immunohistochemistry. *P *<* 0.05

To evaluate the effects of miR-411-5p/3p in a xenograft mouse model, we injected 5 × 10^6^ H1299 cells with either pLenti-miR-411 or pLenti subcutaneously into SCID mice and measured tumor volume weekly. Tumor growth was significantly promoted in the pLenti-miR-411 H1299 cell group 8 weeks post implantation (Fig. 9a). There was also a marked induction in tumor weight (Fig. 9b) and tumor images were shown in Fig. 9c. Moreover, miR-411-5p/3p expression was significantly upregulated (Fig. 9d), and SPRY4 and TXNIP protein expression was downregulated in tumor tissues of the pLenti-miR-411 group (Fig. 9e). We also determined the expression of cadherins relative to EMT. Data revealed that E-cadherin was downregulated, whereas N-cadherin showed the inverse. Meanwhile, EGFR expression and AKT phosphorylation were upregulated (Fig. 9e). Immunohistochemistry assay showed that SPRY4 and E-cadherin were reduced in the pLenti-miR-411 tumor tissues, whereas Ki67 and N-cadherin showed the reverse (Fig. 9f). Collectively, these findings demonstrated that miR-411-5p/3p could promote tumor growth through the repression of SPRY4 and might induce tumor metastasis.

## Discussion

**Fig. 10:**
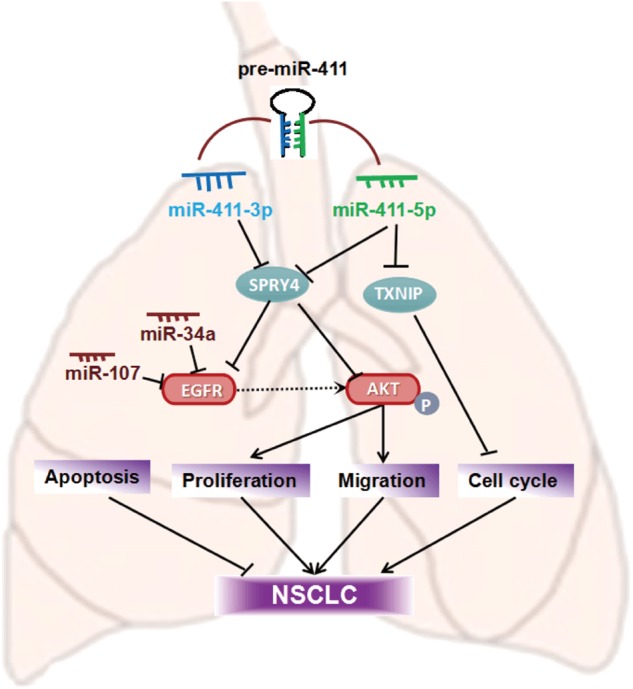
A diagram of the functions of miR-411 in NSCLC

In this study, we noticed that miR-411-5p/3p were upregulated in NSCLC tissues and cells. Overexpression of miR-411-5p/3p promoted tumorigenesis in vitro. miR-411-5p, especially, showed prominent expression level in NSCLC tissues and cells, and had more crucially roles in cell proliferation, apoptosis, and migration. Moreover, miR-411-5p/3p could promote tumor growth and metastasis through downregulation of E-cadherin and upregulation of N-cadherin. *SPRY4* is a common target of miR-411-5p and miR-411-3p. Acting as an oncogene within this regulation network, miR-411-5p/3p increased cell proliferation and migration, while decreased apoptosis in NSCLC (Fig. 10). These results suggested that as a target, *SPRY4* is involved in the oncogenic functions of miR-411-5p/3p in NSCLC.

In lung cancer, SPRY4 is a promising target to interdict the progress of NSCLC therapy engaged in the progression of EGFR-mediated ovarian cancer and enhances melanoma cell motility and treatment of gastrointestinal tumors [12, 31]. Moreover, SPRY4 regulates various signals negatively in angiogenesis, selectively suppressing angiogenic signals independent of Ras in the tumor microenvironment [32]. Also, it is reported to inhibit the mitogen-activated protein kinase (MAPK) signaling pathway. The high SPRY4 expression often impedes cell growth of NSCLC and induces a reversal of EMT characteristic of tumor cells [16]. Herein, we found it interacted with miR-411 and EGFR. SPRY4 inhibited EGFR expression in NSCLC, a critical factor in lung cancer-targeted therapy (Fig. 10). We also found that miR-107 [21] and miR-34a [33] directly target EGFR. These indicate the axis of miR-411/SPRY4/EGFR can also be regulated by other miRNAs such as miR-34a and miR-107. The miRNA regulator network in lung cancer still needs further study.

In addition, miR-411-5p was reduced by transforming growth factor (TGF)-β1 [34] in rhabdomyosarcoma (RMS); thus, the self-regulated loop between TGF-β1/miR-411-5p/SPRY4 and MAPK was formed, and not only activated p38/MAPK phosphorylation but also promoted apoptosis and myogenic differentiation via direct downregulation of SPRY4 in RMS [35]. Furthermore, it impeded cell proliferation and metastasis by directly targeting GRB2 and participated in GRB2-SOS-Ras signaling pathway [36]. Moreover, miR-411 targeted specificity protein 1 in breast cancer [37]. Acting as a cluster, miR-411 and miR-379 inhibited the invasion of malignant pleural mesothelioma cells and appeared to decrease drug resistance (vorinostat (SAHA) and pemetrexed (PEM)) by directly targeting interleukin (IL)-18 [38]. On the other hand, miR-411-5p as an oncogene targeted FOXO1 and ITCH to promote cell cycle process and proliferation in NSCLC [39] and hepatocellular carcinoma [40]. Through upregulating CCND1 and downregulating p27 and p21 expression, miR-411-5p induced A549 and Calu-3 NSCLC cell cycle progress [39]. The aberrant miR-411 expression in the serum of breast cancer patients makes it a biomarker, showing a negative association with breast cancer stage [41]. In osteoarthritis (OA), miR-411 was repressed by IL-1β treatment in OA cartilage, directly targeted MMP-13 to inhibit chondrocyte cell proliferation, and increased expression of type II and type IV collagen [42]. Meanwhile, AKT signaling regulates transcriptional competence and effects miRNA expression [43], such as in NSCLC [44]. Our present results also showed that miR-411-5p/3p induced NSCLC progress. However, whether or not the effect of miR-411-5p/3p on cell cycle is cell specific requires to be further studied.

In conclusion, our findings suggested that miR-411-5p/3p could promote lung carcinogenesis and the tumor suppressor gene *SPRY4* was regulated by miR-411-5p/3p via a direct posttranscriptional mechanism. The miR-411-5p/3p-SPRY4-AKT axis may function as promising therapeutic targets in NSCLC.

## Materials and methods

### Tissue samples and ethics statement

Human lung cancer tissues were obtained from Shanghai Chest Hospital with the patients informed and approved by Ethics Committee of the hospital in accordance with international standards. The detailed information of all tissues is listed in Supplementary Table S1.

### Cell culture and transfection

H1299 and HBE cells were from American Type Culture Collection (Manassas, VA, USA). A549, SPC-A1, PC-9, 95-D, and HEK293T cell lines were from China Academy of Sciences (Shanghai, China).

H1299, 95-D, and HBE cells were maintained in RPMI-1640 medium (Gibco, Gaithersburg, MD, USA) with 10% fetal bovine serum (FBS, HyClone Laboratories, Logan, UT, USA), whereas A549, SPC-A1, PC-9, and HEK293T cells in Dulbecco’s modified Eagle’s medium (Gibco) with 10% FBS in humidified cell incubator, 5% CO_2_ at 37 °C.

Cells were transiently transfected with Lipofectamine 2000 (Invitrogen, Carlsbad, CA, USA) and 200 nM of chemically synthesized miR-411-5p/3p inhibitors or a NC inh and siSPRY4 or siNC (Ribobio, Guangzhou, China) following the standard protocols. qRT-PCR was used to evaluate the RNA level 24 or 48 h after transfection. All the cell phenotypic experiments were performed within 96 h. Relative sequences are listed in Supplementary Table S3.

### Lentiviral construction and infection

The pre-miR-411 (96 bp) was amplified using H1299 genomic DNA and cloned into the pLenti vector (Invitrogen) after double digestion by *BamH* I and *Xho* I, forming pLenti-miR-411 and sequenced (Sangon Biotech, Shanghai, China). Lentiviral particles were collected at 48 and 72 h from HEK293T cells following co-transfection using pLenti or pLenti-miR-411 vector with packaging plasmids (psPAX2 and pMD2G) and saved at 4 °C. The long-term storage environment should be at − 80 °C.

Infection of H1299 and SPC-A1 cells were performed at 50% density for 5 h with pLenti or pLenti-miR-411 viral particles and green fluorescence positive cells were sorted by flow cytometry (Beckman Coulter, Inc., Brea, CA, USA) to establish pLenti/pLenti-miR-411 stably transfected H1299 and SPC-A1 cells (pLenti/pLenti-miR-411 H1299, and SPC-A1), followed by enlarge cultivation for further experiments.

### RNA extraction and qRT-PCR

Total RNA was isolated with Trizol (Sangon Biotech) following standard instruction. The miRNA cDNA library was established using SuperMixQuantiMir cDNA Kit (Transgen Biotec, Beijing, China) following reverse transcription of the RNAs using M-MLV RTase cDNA Synthesis Kit (TaKaRa, Dalian, China). SYBR Green PCR mix (TaKaRa) was used in qRT-PCR for quantification of miRNA and mRNA expression with U6 snRNA and 18S RNA as the internal controls, respectively. Relative quantification (2^−^^ΔΔCT^) was used for results analysis. PCR primers are listed in Supplementary Table S2.

### Western blotting

Western blotting as well as total protein extraction and quantification were performed following procedures as we described previously [33]. Rabbit anti-SPRY4 or anti-TXNIP (1:1000, Abcam, Cambridge, UK), anti-E-cadherin/N-cadherin/p-AKT1/AKT1/EGFR, and anti-GAPDH antibodies (1:1000, Cell Signaling Technology, Danvers, MA, USA) were used, respectively, as the first antibody, and horseradish peroxidase 1:10,000, Transgene Biotech, Beijing, China)-conjugated goat-anti-rabbit antibody were used as the secondary antibody.

### Cell proliferation assay

Cell proliferation was assessed with CCK-8 assay (Dojindo, Japan) every 24 h until 96 h. Absorbance was measured 2.5 h after incubation with 100 μL FBS-free medium containing 5% CCK-8.

### Cell apoptosis assay

Cells were treated with actinomycin D (5 μg/mL) for 12 h and then stained for Annexin-V APC/7AAD (BioLegend, San Diego, USA) or PI/FITC (BD Pharmingen, New York, USA). Flow cytometry was then used for apoptosis assay.

### Would-healing assay and transwell assay

Wound-healing assay and transwell assay were performed following protocols as we described previously [33]. For wound-healing assay, cell locations were recorded with the gap measured at 0 h and 48 h, respectively, whereas in transwell assay, cells were fixed, stained, and photographed 48 h post incubation, and then the cell numbers were counted.

### 3′-UTR cloning and validation

The SPRY4 mRNA 3′-UTR containing the miR-411-5p/3p-binding sites was sub-cloned into the pGL3 miReport vector (Promega, Madison, WI, USA) and named as pGL3-SPRY4-3′-UTR, whereas vector containing the mutated SPRY4 3′-UTR were named as pGL3-SPRY4-3′-mUTR. The sequence of the recombinant vectors was confirmed by sequencing (Supplementary Table S2).

HEK293T cells were transiently co-transfected with pGL3-SPRY4-3′-UTR or pGL3-SPRY4-3′-mUTR, miR-411-5p/3p or NC mimic, and pRL vector (Promega). The luciferase activity was assessed 48 h post transfection.

### Expression vector construction

The full-length human SPRY4 coding region (primers sequences in Supplementary Table S2) was amplified using DNA polymerase Phanta (Vazyme Biotech, Nanjing, China) and sub-cloned into the pcDNA3.1 (-) vector (5427 bp) with *Xba* I and *EcoR* I restriction sites, and designated as pcDNA3.1-SPRY4 followed by sequencing (Supplementary Table S2). Then followed transfection pLenti/pLenti-miR-411 H1299 cells with the above-mentioned vectors (1 μg/mL). The SPRY4 expression was determined 24 h post transfection by qRT-PCR and western blotting.

### Xenograft tumor assay

Female SCID mice (6–8 weeks old, SLRC Laboratory Animal Center, Shanghai, China) were fed in specific pathogen-free environment and randomly assigned to one of two groups. pLenti or pLenti-miR-411 H1299 cells (5 × 10^6^) were subcutaneously implanted into mice at the right flank. Tumor volume was calculated every week using the formula (volume = length × width^2^/2). The mice were killed 8 weeks post injection. The tumors were weighed and used for further experiments. Animal study followed rules of the institutional Animal Care and Use Committee of Shanghai University (Shanghai, China).

### Immunohistochemistry assay

Tissue samples were embedded in paraffin before deparaffinization and rehydration followed by incubation with primary antibodies against SPRY4, Ki67, E-cadherin, and N-cadherin (1:500), and secondary antibody, followed by staining and photographing.

### Statistical analysis

Results were showed as group means ± SEM. The Student’s *t*-test was used to analyze two groups’ comparisons. *p* < 0.05 means statistically significant.

## Electronic supplementary material

Supplement 1

Supplement 2

Supplement 3

Supplementary Figure legends

Supplymental table S1

Supplymental table S2

Supplymental table S3

Cell line authentication
