# Supplementary figures and images for: Oncogenic microRNA-411 promotes lung carcinogenesis by directly targeting suppressor genes SPRY4 and TXNIP
Source: Oncogene. 2018 Nov 2;38(11):1892–904. doi: 10.1038/s41388-018-0534-3 (PMC6475890; doi:10.1038/s41388-018-0534-3)

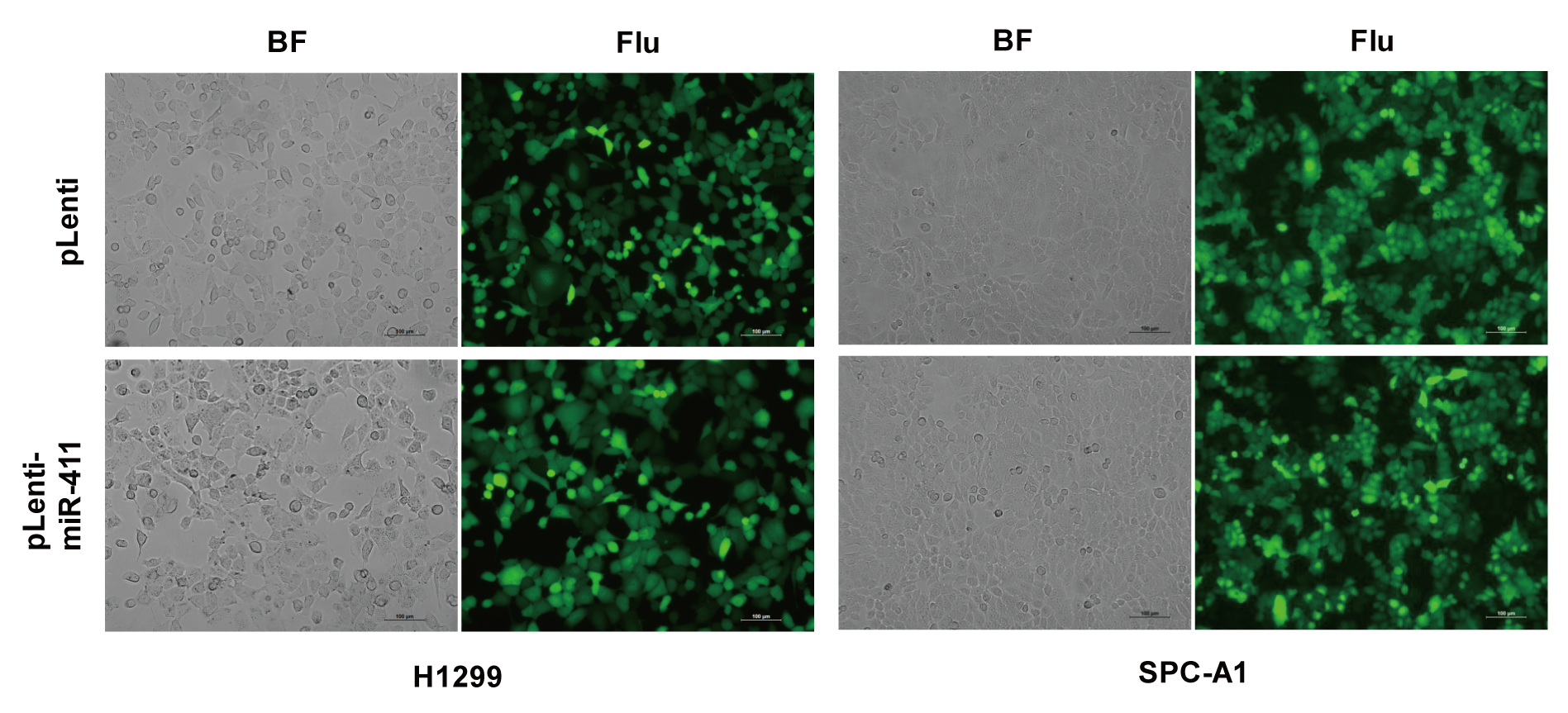

Supplement: Supplementary file 1 — Supplement 1 [file 41388_2018_534_MOESM1_ESM.tif]

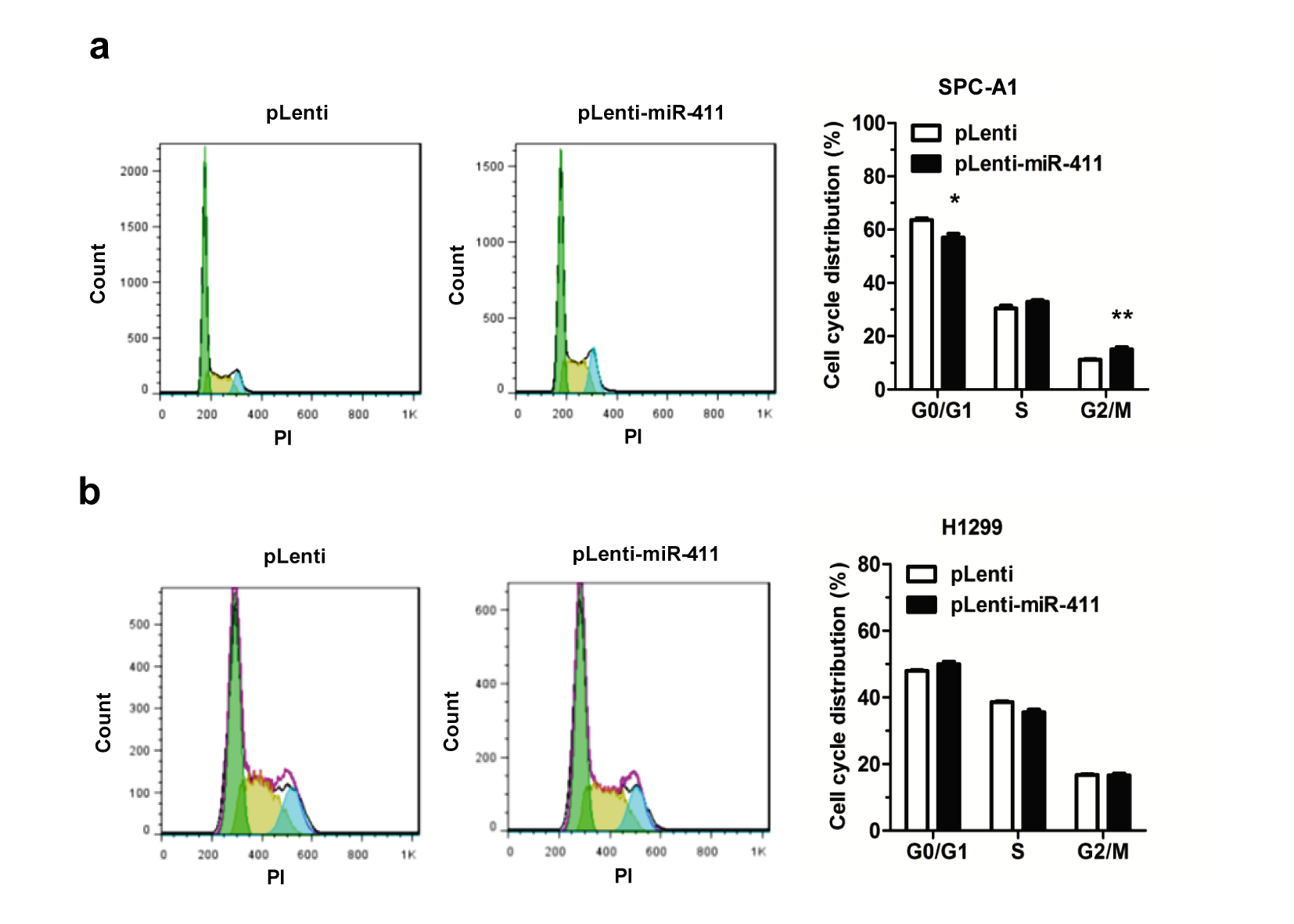

Supplement: Supplementary file 2 — Supplement 2 [file 41388_2018_534_MOESM2_ESM.tif]

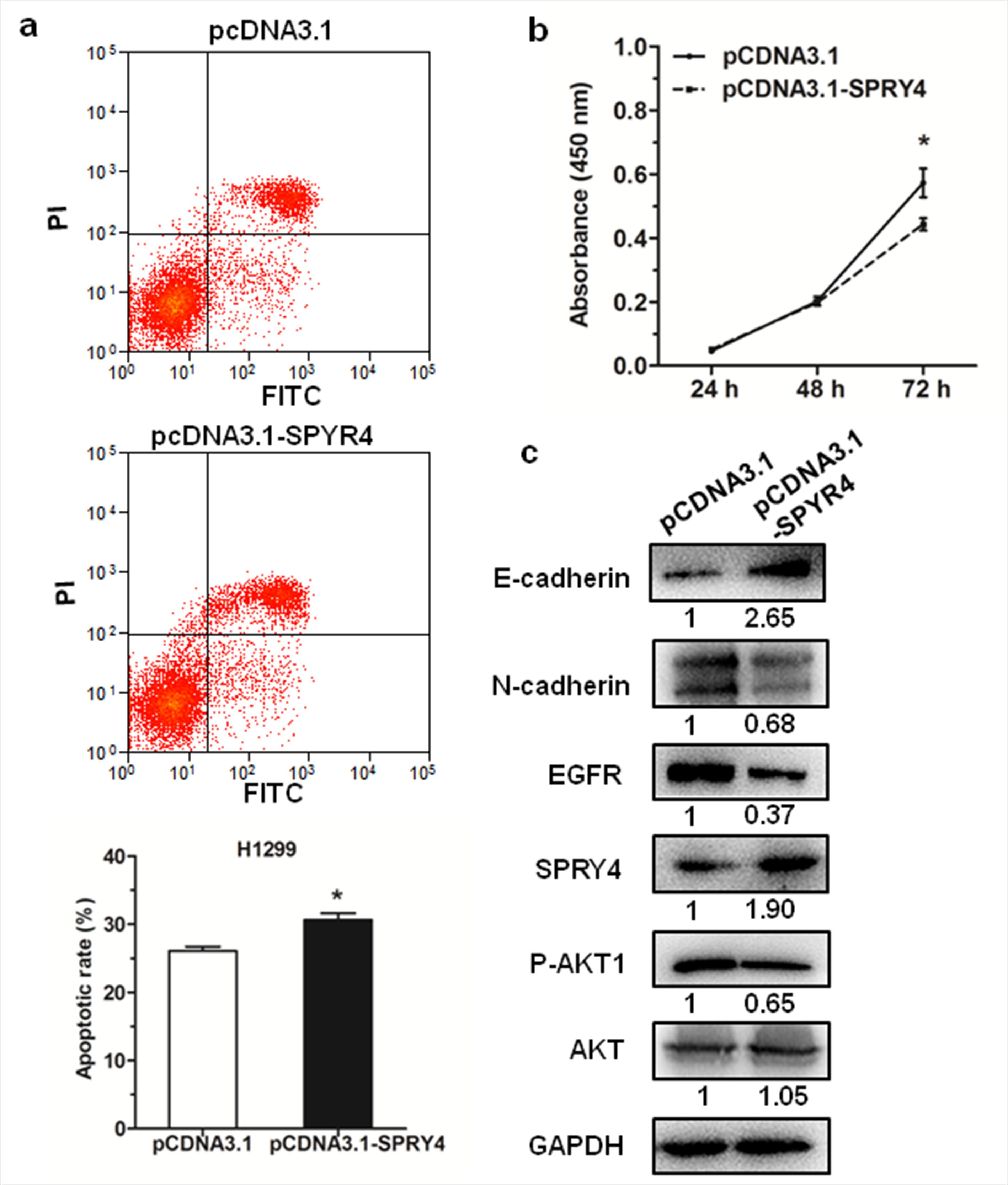

Supplement: Supplementary file 3 — Supplement 3 [file 41388_2018_534_MOESM3_ESM.tif]
