# Supplementary material for: Oncogenic microRNA-411 promotes lung carcinogenesis by directly targeting suppressor genes SPRY4 and TXNIP
Source: Oncogene. 2018 Nov 2;38(11):1892–904. doi: 10.1038/s41388-018-0534-3 (PMC6475890; doi:10.1038/s41388-018-0534-3)
Supplement: Supplementary file 4 — Supplementary Figure legends [file 41388_2018_534_MOESM4_ESM.docx]

**Supplementary Figure legends**

**Supplementary Figure 1.** **The positive pLenti-miR-411 H1299 cells and SPC-A1 cells were shown by imaging with inverted fluorescence microscope.**

**Supplementary Figure 2.** **MiR-411 can effect cell-cycle progression in NSCLC.**

1. The cell cycle distributions of pLenti-miR-411 SPC-A1 cells were detected by flow cytometry.
2. The cell cycle distributions of pLenti-miR-411 H1299 cells were detected by flow cytometry.

**P*<0.05 and **P<0.01.

**Supplementary Figure 3. Over-expression of SPRY4 induced cell apoptosis and proliferation suppression in H1299 cells.**

**(a)** Apoptosis rates were analyzed by flow cytometry in H1299 cells transfected with pcDNA3.1-SPRY4.

**(b)** The proliferation of H1299 cells transfected with pcDNA3.1-SPRY4 or pcDNA3.1 were measured by CCK-8 assay.

**(c)** The expression of E-cadherin, N-cadherin, EGFR, SPRY4 and AKT proteins was detected by western blot in the H1299 cells transfected with pcDNA3.1-SPRY4 or pcDNA3.1. **P* < 0.05, ***P* < 0.01, ****P* < 0.001.
