# Supplementary material for: Oncogenic microRNA-411 promotes lung carcinogenesis by directly targeting suppressor genes SPRY4 and TXNIP
Source: Oncogene. 2018 Nov 2;38(11):1892–904. doi: 10.1038/s41388-018-0534-3 (PMC6475890; doi:10.1038/s41388-018-0534-3)
Supplement: Supplementary file 5 — Supplymental table S1 [file 41388_2018_534_MOESM5_ESM.doc]

**Table S1** Information for clinical samples

| Number | Gender | Stage | Histopathologic classification |
| --- | --- | --- | --- |
| 1 | Male | IA | Adenocarcinoma |
| 2 | Female | IB | Adenocarcinoma |
| 3 | Female | IIIA | Adenocarcinoma |
| 4 | Male | IIIA | Adenocarcinoma |
| 5 | Male | IIIA | Adenocarcinoma |
| 6 | Female | IIIA | Adenocarcinoma |
| 7 | Male | IV | Adenocarcinoma |
| 8 | Female | IV | Adenocarcinoma |
| 9 | Male | IA | Adenocarcinoma |
| 10 | Male | IIA | Squamous carcinoma |
| 11 | Male | IIIA | Squamous carcinoma |
| 12 | Male | IIIA | Squamous carcinoma |
| 13 | Male | IIIA | Squamous carcinoma |
| 14 | Male | IB | Adenocarcinoma |
| 15 | Female | IIB | Squamous carcinoma |
| 16 | Male | IIIA | Adenocarcinoma |
| 17 | Male | IIA | Squamous carcinoma |
| 18 | Male | IB | Adenocarcinoma |
| 19 | Male | IA | Squamous carcinoma |
| 20 | Male | IIIA | Adenocarcinoma |
| 21 | Female | IIIA | Adenocarcinoma |
| 22 | Male | IIB | Adenocarcinoma |
| 23 | Female | IIB | Adenocarcinoma |
| 24 | Male | IA | Adenocarcinoma |
| 25 | Male | IA | Adenocarcinoma |
| 26 | Male | IA | Squamous carcinoma |
| 27 | Female | IIIB | Adenocarcinoma |
| 28 | Female | IIIB | Adenocarcinoma |
| 29 | Female | IA | Adenocarcinoma |
| 30 | Male | IIIA | Adenocarcinoma |
| 31 | Female | IIB | Adenocarcinoma |
| 32 | Female | IIB | Adenocarcinoma |
| 33 | Female | IIIB | Adenocarcinoma |
