# Supplementary material for: Oncogenic microRNA-411 promotes lung carcinogenesis by directly targeting suppressor genes SPRY4 and TXNIP
Source: Oncogene. 2018 Nov 2;38(11):1892–904. doi: 10.1038/s41388-018-0534-3 (PMC6475890; doi:10.1038/s41388-018-0534-3)
Supplement: Supplementary file 6 — Supplymental table S2 [file 41388_2018_534_MOESM6_ESM.doc]

**Table S2** Primer sequences used for amplification

| Name | Usage | Sequence ( 5‵- 3‵) |
| --- | --- | --- |
| 18S RNA | qPCR forward | AGGAATTCCCAGTAAGTGCG |
|  | qPCR reverse | GCCTCACTAAACCATCCAA |
| U6 snRNA | qPCR forward | CTCGCTTCGGCAGCACA |
|  | qPCR reverse | AACGCTTCACGAATTTGCGT |
| SPRY4 | qPCR forward | CGGAAAATACAGAGACCACC |
|  | qPCR reverse | GGACCCTGAAAAAAAGCC |
| SPRY4-  3′-UTR-1 | PCR forward  PCR reverse | GCTCTAGA CTTGCCTTGCTTTCTCTT  CGGAATTC GCAGTCCCGTGTATATTTAAC |
| SPRY4-  3′-UTR-2 | PCR forward  PCR reverse | GCTCTAGA ACACACATTCAGCCAACCA  CGGAATTC GCAGACATCCATCAAGCA |
| SPRY4-  3′-mUTR-1 | PCR forward  PCR reverse | AGACAAGTTTTGTAAGAGTTCTTCTGTTTGT  CAAAACTTGTCTTTCTAAAATAAATACTAT |
| SPRY4-  3′-mUTR-2 | PCR forward  PCR reverse | CGATTAAGAAAAGCTGTGTATACACACACACACAC  CAGCTTTTCTTAATCGACTCTCCACAGAAC |
| pCDNA3.1(-)-SPRY4 | PCR forward  PCR reverse | GCTCTAGAATGGAGCCCCCGATCCCACAGA  CGGAATTCTCAGAAAGGCTTGTCGGGCCTGC |
