# Supplementary material for: Oncogenic microRNA-411 promotes lung carcinogenesis by directly targeting suppressor genes SPRY4 and TXNIP
Source: Oncogene. 2018 Nov 2;38(11):1892–904. doi: 10.1038/s41388-018-0534-3 (PMC6475890; doi:10.1038/s41388-018-0534-3)
Supplement: Supplementary file 7 — Supplymental table S3 [file 41388_2018_534_MOESM7_ESM.doc]

**Table S3** Sequences of the NC siRNA and siRNA for SPRY4

| Name | Target mRNA | Sequence ( 5‵- 3‵) |
| --- | --- | --- |
| siSPRY4 (-1) | SPRY4 (H1299) | CAGCACATCCTCTGACCAA |
| siSPRY4 (-2) | SPRY4 (SPCA-1) | CTGCACGAATGAGGACGAT |
| siSPRY4 (-3) | SPRY4 | TGTGGAGAATGACTACATA |
