# Supplementary material for: Oncogenic microRNA-411 promotes lung carcinogenesis by directly targeting suppressor genes SPRY4 and TXNIP
Source: Oncogene. 2018 Nov 2;38(11):1892–904. doi: 10.1038/s41388-018-0534-3 (PMC6475890; doi:10.1038/s41388-018-0534-3)
Supplement: Supplementary file 8 — Cell line authentication [file 41388_2018_534_MOESM8_ESM.pdf]

SHANGHAI BIOWING APPLIED BIOTECHNOLOGY Co. LTD

Cell Line Authentication – STR Profiling

Sample Type: Cell Line

Sample from: Shanghai University

Testing Method: STR Genotyping

Report Time: December 14, 2016

COMPANY STATEMENT

1. THIS REPORT IS ONLY RESPONSIBLE FOR THE SAMPLES ANALYZED.
2. THE TESTING RESULTS AND THE ORGANIZATION NAME WILL NOT BE USED FOR ADVERTISEMENT, COMMERCIAL EXHIBITIONS, COMMERCIAL PERFORMANCE AND OTHER COMMERCIAL ACTIVITIES.
3. OBJECTIONS SHOULD BE RAISED WITHIN FIFTEEN DAYS AFTER THE RECEIPT OF THIS REPORT.
4. THE PAPER REPORT WITH CONTENT ALTERING, ADDING OR WITHOUT THE STAMPED SEAL OF THE COMPANY ARE INVALID.

Testing Company: Shanghai Biowing Applied Biotechnology Co. Ltd

Address: Room 4F, 8th Buiding, Guiguo Garden, NO.471 Guiping Road, Caohejing Development Zone, Shanghai

Tel: +86-021-33559491

Contact: YiQun Chen

E-mail: biowing@vip.163.com

Cell Line Authentication – STR Profiling Report

Sample code

Table 1. Sample Code

Customer's code	Company Code
01	20161206-01
02	20161206-02
03	20161206-03
04	20161206-04

Sample Number:4

Sample Type: Cell line

Testing Type: STR

Sample From: Shanghai University

Testing Method:

DNA was extracted by a commercial kit from CORNING (AP-EMN-BL-GDNA-250G). The twenty STRs including Amelogenin locus were amplified by six multiplex PCR and separated on ABI 3730XL Genetic Analyzer. The signals were then analyzed by the software GeneMapper.

Data Interpretation:

Cell lines were authenticated using Short Tandem Repeat (STR) analysis as described in 2012 in ANSI Standard (ASN-0002) by the ATCC Standards Development Organization (SDO) and in Capes-Davis et al., Match criteria for human cell line authentication: Where do we draw the line? Int J Cancer.2013;132(11):2510-9.

Test Results:

1. Result

Table 2. Matching information on the cell lines

Sample Code	Multi-allele	Cell line matched	Cell Bank	EV	Percentage
20161206-01	No	SPC-A1	CRC	1	9/9
20161206-02	No	NCI-H1299	DSMZ	0.947	9/9
20161206-03	No	A549	DSMZ	1	9/9
20161206-04	No	PC-9	DSMZ	1	9/9

- **Multi-allele means some STR contain more than two loci.**

2. Sample Description

- 20161206-01: The DNA of the cell lines found to perfect match the type of cell lines in a cell line retrieval, CRC database shows that cells called **SPC-A1，** corresponding to the cell number **80**. No multiple alleles were found in this cell line.
- 20161206-02: The DNA of the cell lines found to basic match the type of cell lines in a cell line retrieval, DSMZ database shows that cells called **NCI-H1299,** corresponding to the cell number **CRL-5803**. No multiple alleles were found in this cell line.
- 20161216-03: The DNA of the cell lines found to perfect match the type of cell lines in a cell line retrieval, DSMZ database shows that cells called **A549,** corresponding to the cell number **CCL-185,**. No multiple alleles were found in this cell line.
- 20161216-04: The DNA of the cell lines found to perfect match the type of cell lines in a cell line retrieval, GNE database shows that cells called **PC-9,** corresponding to the cell number **0.** No multiple alleles were found in this cell line.

3. Genotyping Result

Table 3. STR and Amelogenin Genotyping Results of Cell line 20161216-01

Marker	Sample				Cell Bank information		
	Allele1	Allele2	Allele3	Allele4	Allele1	Allele2	Allele3
D5S818	11	12			11	12	
D13S317	12	13.3			12	13.3	
D7S820	8	12			8	12	
D16S539	9	10			9	10	
VWA	16	18			16	18	
TH01	7	7			7	7	
AMEL	X	X			X	X	
TPOX	8	12			8	12	
CSF1PO	9	10			9	10	
D12S391	20	25					

FGA	21	21					
D2S1338	17	17					
D21S11	27	28					
D18S51	16	16					
D8S1179	12	12					
D3S1358	15	18					
D6S1043	18	19					
PENTAE	7	17					
D19S433	13	14					
PENTAD	8	15					

Table 4. STR and Amelogenin Genotyping Results of Cell line 20161216-02

**Marker**	样本				细胞库信息		
	**Allele1**	**Allele2**	**Allele3**	**Allele4**	**Allele1**	**Allele2**	**Allele3**
D5S818	11	11			11	11	
D13S317	12	12			12	12	
D7S820	10	10			10	10	
D16S539	12	13			12	13	
VWA	16	18			16	17	18
TH01	6	9.3			6	9.3	
AMEL	X	X			X	X	
TPOX	8	8			8	8	
CSF1PO	12	12			12	12	
D12S391	21	22					
FGA	20	20					
D2S1338	23	24					
D21S11	32.2	32.2					
D18S51	16	16					
D8S1179	10	13					
D3S1358	17	17					
D6S1043	11	13					
PENTAE	11	11					
D19S433	14	14					
PENTAD	13	13					

Table 5. STR and Amelogenin Genotyping Results of Cell line 20161216-03

**Marker**	样本				细胞库信息		
	**Allele1**	**Allele2**	**Allele3**	**Allele4**	**Allele1**	**Allele2**	**Allele3**
D5S818	11	11			11	11	
D13S317	11	11			11	11	
D7S820	8	11			8	11	
D16S539	11	12			11	12	
VWA	14	14			14	14	
TH01	8	9.3			8	9.3	
AMEL	X	X			X	X	
TPOX	8	11			8	11	
CSF1PO	10	12			10	12	

D12S391	18	18					
FGA	23	23					
D2S1338	24	24					
D21S11	29	29					
D18S51	14	17					
D8S1179	13	14					
D3S1358	16	16					
D6S1043	11	13					
PENTAE	7	11					
D19S433	13	13					
PENTAD	9	9					

Table 6. STR and Amelogenin Genotyping Results of Cell line 20161216-04

Marker	样本				细胞库信息		
	**Allele1**	**Allele2**	**Allele3**	**Allele4**	**Allele1**	**Allele2**	**Allele3**
D5S818	11	11			11	11	
D13S317	8	8			8	8	
D7S820	10	11			10	11	
D16S539	9	9			9	9	
VWA	17	17			17	17	
TH01	7	7			7	7	
AMEL	X	X			X	X	
TPOX	11	11			11	11	
CSF1PO	11	11			11	11	
D12S391	18	18					
FGA	23	25					
D2S1338	19	20					
D21S11	29	30					
D18S51	15	15					
D8S1179	11	15					
D3S1358	16	16					
D6S1043	13	19					
PENTAE	11	15					
D19S433	13	15.2					
PENTAD	9	13					

Others:

1. Genotyping Strategy and Site Distribution

Attached Table. Experimental Strategy and Sites

	**Strategy 1**	**Strategy 2**	**Strategy 3**	**Strategy 4**	**Strategy 5**	**Strategy 6**
1	TH01	AMEL	TPOX	D3S1358	PENTAE	D19S433
2	D12S391	D5S818	VWA	D13S317		PENTAD
3	D7S820	D2S1338	D8S1179	D6S1043		

4	CSF1PO	D21S11		D16S539		
5	FGA	D18S51				

The allele match algorithm compares the 8 core loci plus amelogenin only, even though alleles from all loci will be reported when available.

2. DSMZ tools was used to carry on the cell line comparison, which contains 2455 cell lines STR data from ATCC, DSMZ, JCRB ,ECACC，GNE and RIKEN databases. If the cell is not included in the above cell library, users need to compared with other databases.

Technician:Menglu Shen
Check: Yang Bai
Person in Charge: Yiqun Chen

Issue date: December 14, 2016

细胞遗传质量鉴定检测

Cell Line Authentication Service

STR 基因型检测报告

送检单位：上海大学生命学院

检品名称：细胞系

委托单位：上海翼和应用生物技术有限公司

报告日期：2018-05-07

上海翼和应用生物技术有限公司 联系地址：上海市漕河泾开发区桂平路 471 号桂果园 8 号楼 4F 室
网址：http://www.biowing.com.cn/邮箱：biowing@vip.163.com 服务热线：021-33559491

报 告 说 明

1. 本报告只对送检的来样负责。
2. 检验报告上的检验结果和检验单位名称，未经同意不得用于广告、评优及 商业宣传。
3. 对本报告有异议，请于收到报告之日起十五日内以书面方式提出，逾期不 予受理。
4. 对纸质检验报告涂改、增删，或未加盖检验单位印章的复印件均无效。

上海翼和应用生物技术有限公司 联系地址：上海市漕河泾开发区桂平路 471 号桂果园 8 号楼 4F 室
网址：http://www.biowing.com.cn/邮箱：biowing@vip.163.com 服务热线：021-33559491

样 品 信 息

样品编号：

客户样本编号	公司编号
95-D	20180502-02

样品数量： 1

样品性状： 细胞系

检测项目： STR

送检单位： 上海大学生命学院

检测方法： 用 Axygen 的基因组抽提试剂盒提取DNA，采用 20- STR 扩增方案扩增，在 ABI 3730XL 型遗传分析仪上对 STR 位点和性别基因Amelogenin 进行检测。

检 测 结 果

(一) 检验基本情况

	多等位基因	匹配细胞系	细胞库	EV 值	匹配说明
20180502-02	无	95-D	CRC	1.00	完全匹配

样本基因型检验结果

- 多等位基因指三等位及以上基因现象。
- 本次检测各细胞分型结果良好。

(二) 各样本描述

- 20180502-02：该株细胞 DNA 分型在细胞系检索中**没有找到匹配**的细胞系，CRC 数据库显示细胞名为 **95-D**，细胞号对应 **3131C0001000700061**。本次检测在该细胞系中**没有发现多等位基因**。

  备注：待测细胞系与收录于 ATCC, DSMZ, JCRB 和 RIKEN 数据库的细胞系 STR 数据进行比对，未收录于以上细胞库的细胞系将无法匹配。

（三）样本分型结果

细胞 20180502-02 的 STR 位点和Amelogenin 位点的基因分型结果						
Loci	送检细胞STR 信息			细胞库细胞STR 信息		
	送检细胞名：95-D			细胞库细胞名：95-D		
	Allele1	Allele2	Allele3	Allele1	Allele2	Allele3
D5S818	11	11		11	11	
D13S317	12	12		12	12	
D7S820	9	11		9	11	
D16S539	13	13		13	13	
VWA	18	18		18	18	
TH01	7	7		7	7	
AMEL	X	Y		X	Y	
TPOX	9	11		9	11	
CSF1PO	12	12		12	12	
D12S391	19	20				
FGA	19	19				
D2S1338	18	18				
D21S11	29	29				
D18S51	14	14				
D8S1179	13	15				
D3S1358	16	16				
D6S1043	11	19				
PENTAE	12	16				
D19S433	13	15.2				
PENTAD	6	6				

其 他 说 明

(一) 分型方案及位点分布

	方案 1	方案 2	方案 3	方案 4
1	TH01	TPOX	D3S1358	AMEL
2	D12S391	VWA	D13S317	D5S818
3	D7S820	D8S1179	D6S1043	D2S1338
4	CSF1PO	PENTAD	D16S539	D21S11
5	FGA		D19S433	D18S51
6	PENTAE			

实验方案及位点

(二) STR 数据库比对

本公司采用DSMZ tools 进行细胞系比对，其中包含来自于 ATCC, DSMZ, JCRB 和 RIKEN 数据库的 2455 个细胞系 STR 数据。如果待检测细胞未收录于以上细胞库或这是自行建立的新细胞系将无法进行比对，用户需根据细胞分型结果自行与其他数据库进行比对。

(三) 文献引用参考

1．Authentication testing of HEK 293T and HeLa cell lines have been performed by Shanghai Biowing Applied Biotechnology Co.,Ltd via STR profiling. STR profiles match the standards recommended for HEK 293T and HeLa cell lines authentication

2．AGS, NCI-N87, HGC-27 and HEK293 were STR-authenticated on Dec. 8, 2015 by Shanghai Biowing Applied Biotechnology Co. LTD, Shanghai, China

主要实验人员：张佳男

复核人: 龚礼瑾

负责人： 陈轶群

签发日期：2018 年 05 月 07 日

上海翼和应用生物技术有限公司 联系地址：上海市漕河泾开发区桂平路 471 号桂果园 8 号楼 4F 室
网址：http://www.biowing.com.cn/邮箱：biowing@vip.163.com 服务热线：021-33559491

细胞遗传质量鉴定检测

Cell Line Authentication Service

STR 基因型检测报告

送检单位：上海大学生命学院
检品名称：细胞系
委托单位：上海翼和应用生物技术有限公司
报告日期：2018-05-07

报 告 说 明

1. 本报告只对送检的来样负责。
2. 检验报告上的检验结果和检验单位名称，未经同意不得用于广告、评优及商业宣传。
3. 对本报告有异议，请于收到报告之日起十五日内以书面方式提出，逾期不予受理。
4. 对纸质检验报告涂改、增删，或未加盖检验单位印章的复印件均无效。

上海翼和应用生物技术有限公司 联系地址：上海市漕河泾开发区桂平路 471 号桂果园 8 号楼 4F 室
网址：http://www.biowing.com.cn/邮箱：biowing@vip.163.com 服务热线：021-33559491

样 品 信 息

样品编号：

客户样本编号	公司编号
293T	20180502-03

样品数量：3

样品性状：细胞系

检测项目：STR

送检单位：上海大学生命学院

检测方法：用 Axygen 的基因组抽提试剂盒提取 DNA，采用 20- STR 扩增方案扩增，在 ABI 3730XL 型遗传分析仪上对 STR 位点和性别基因 Amelogenin 进行检测。

上海翼和应用生物技术有限公司 联系地址：上海市漕河泾开发区桂平路 471 号桂果园 8 号楼 4F 室
网址：http://www.biowing.com.cn/邮箱：biowing@vip.163.com 服务热线：021-33559491

检 测 结 果

(一) 检验基本情况

	多等位基因	匹配细胞系	细胞库	EV 值	匹配说明
20180502-03	有	293T 等	DSMZ	0.85	基本匹配

样本基因型检验结果

- 多等位基因指三等位及以上基因现象。
- 本次检测各细胞分型结果良好。

(二) 各样本描述

- 20180502-03：该株细胞 DNA 分型在细胞系检索中找到**基本匹配**的细胞系，DSMZ 数据库显示细胞名为 **293T**，细胞号对应 **CRL-11268** 。本次检测在该细胞系中**发现多等位基因**。**备注：**待测细胞系与收录于 ATCC, DSMZ, JCRB 和 RIKEN 数据库的细胞系 STR 数据进行比对，未收录于以上细胞库的细胞系将无法匹配。

上海翼和应用生物技术有限公司 联系地址：上海市漕河泾开发区桂平路 471 号桂果园 8 号楼 4F 室
网址：http://www.biowing.com.cn/邮箱：biowing@vip.163.com 服务热线：021-33559491

（三）样本分型结果

细胞 20180502-03 的 STR 位点和 Amelogenin 位点的基因分型结果						
Loci	送检细胞 STR 信息			细胞库细胞 STR 信息		
	送检细胞名：293T			细胞库细胞名：293T		
	Allele1	Allele2	Allele3	Allele1	Allele2	Allele3
D5S818	8	9		8	9	
D13S317	11	12		12	14	
D7S820	11	11		11	11	
D16S539	9	12		9	13	
VWA	17	18	19	16	18	19
TH01	7	9.3		7	9.3	
AMEL	X	X		X	X	
TPOX	11	11		11	11	
CSF1PO	11	12	14	11	12	
D12S391	21	22	23			
FGA	22	23				
D2S1338	19	19				
D21S11	27	28				
D18S51	17	18				
D8S1179	11	13				
D3S1358	15	17	18			
D6S1043	10	11				
PENTAE	7	15				
D19S433	16	17	18			
PENTAD	9	10				

上海翼和应用生物技术有限公司 联系地址：上海市漕河泾开发区桂平路 471 号桂果园 8 号楼 4F 室
网址：http://www.biowing.com.cn/邮箱：biowing@vip.163.com 服务热线：021-33559491

其他说明

(一) 分型方案及位点分布

	方案 1	方案 2	方案 3	方案 4
1	TH01	TPOX	D3S1358	AMEL
2	D12S391	VWA	D13S317	D5S818
3	D7S820	D8S1179	D6S1043	D2S1338
4	CSF1PO	PENTAD	D16S539	D21S11
5	FGA		D19S433	D18S51
6	PENTAE			

实验方案及位点

(二) STR 数据库比对

本公司采用 DSMZ tools 进行细胞系比对，其中包含来自于 ATCC, DSMZ, JCRB 和 RIKEN 数据库的 2455 个细胞系 STR 数据。如果待检测细胞未收录于以上细胞库或这是自行建立的新细胞系将无法进行比对，用户需根据细胞分型结果自行与其他数据库进行比对。

(三) 文献引用参考

1．Authentication testing of HEK 293T and HeLa cell lines have been performed by Shanghai Biowing Applied Biotechnology Co.,Ltd via STR profiling. STR profiles match the standards recommended for HEK 293T and HeLa cell lines authentication

2．AGS, NCI-N87, HGC-27 and HEK293 were STR-authenticated on Dec. 8, 2015 by Shanghai Biowing Applied Biotechnology Co. LTD, Shanghai, China

主要实验人员：张佳男

复核人: 龚礼瑾

负责人：陈轶群

签发日期：2018 年 05 月 07 日

细胞 STR 鉴定报告

送样细胞株(Cell lines to be analyzed)：HBE4-E6/E7

实验设计及操作说明(Experimental prodedures)

利用荧光标记扩增产物长度多态分析方法对细胞样本进行 9 个 STR 位点进行分型。设计了 9 对 PCR 引物，组成 2 个 PANEL 用于扩增 9 个多态位点。位点的荧光标记采用引物上标记 5'FAM 荧光标记。引物用在线 Primer3 软件设计（http://frodo.wi.mit.edu/cgi-bin/primer3/primer3_www.cgi）。PCR 产物稀释后取少量与内标标记混匀后直接上 ABI3130xl 进行毛细管电泳，数据文件用 GeneMapper4.0（Appliedbiosystems）来分析。

实验所用试剂和仪器：

HotstarTaq DNA Polymerase	Qiagen
GeneScan™-500 Liz Size Standard	Applied Biosystoms
Hi-Di formamide	Applied Biosystoms
2720 Thermal Cycler	Applied Biosystoms
3130xl Genetic Analyzer	Applied Biosystoms
Centrifuge 5418D	Eppendorf

具体实验操作步骤

1） DNA 样本取 1μl 1% agarose 电泳对其样本呢进行质量检查以及浓度估计，然后根据估计的浓度将样本稀释到工作浓度 5-10ng/μl.

2） PCR 反应

a) PCR 引物

采用公司现有的细胞鉴定的 9 个 STR 引物体系（PANEL 1：TPOX、D16S539、TH01、CSF1PO、D5S818;PANEL 2：Amelo、vWA、D7S820、D13S317）

b) PCR 条件：

A 10μl mixture was prepared for each reaction and included 1x HotStarTaq buffer, 3.0 mM Mg2+, 0.3 mM dNTP, 0.1μM of primer mix,1 U HotStarTaq polymerase (Qiagen Inc.) and 1 μl template DNA.

PCR 程序：

The cycling program was 95ºC for 2 min;

Cinoasia Institute 上海市邯郸路 100 弄 54 甲 102-104；13916639227
复旦大学科技园；shiyong@cinoasia.com

11 cycles of 94℃ for 20 s,

62℃-0.5 ℃ per cycle for 40 s,

68℃ for 2mins;

24cycles of 94℃ for 20 s,

56 ℃ for 30s,

68℃ for 2mins;

60℃ 60 min;

4 ℃ for ever.

4) 取 1μl PCR 产物稀释 80 倍后，取 1ul 与 0.5μl Liz500 SIZE STANDARD，8.5μl Hi-Di 混匀， 95 ℃ 变性 5 分钟后上 ABI3130XL 测序仪

5）ABI3130xl 产生的数据文件用 GeneMapper4.0（Appliedbiosystems）来分析。

6)输出结果采用 EXCEL 进行整理、统计和分析。得出的数据结果在 STR matching analysis（http://www.dsmz.de/fp/cgi-bin/str.html）进行数据库比对，最终得到样本的细胞株鉴定结果表

鉴定结果(Results)

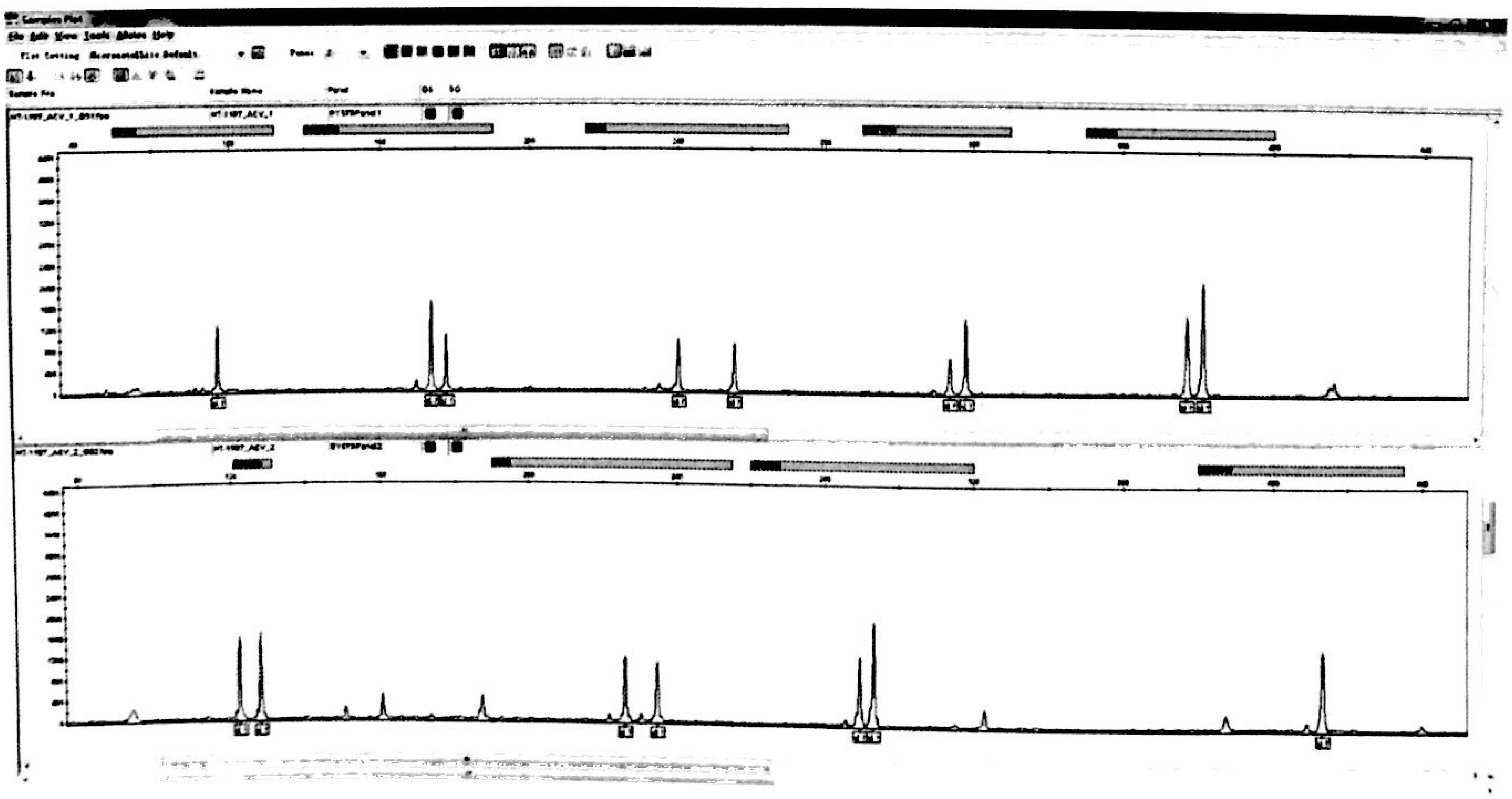

D5S818	D13S317	D7S820	D16S539	vWA	THO1	AM	TPOX	CSF1PO
10,11	11,11	10,11	11,13	14,17	6,8	X,Y	8,8	11,12

数据库比对：

Result of STR matching analysis by your data.

- DSMZ Profile Database -

A graphical presentation is shown at the bottom of this page.

EV	Cell No.	Cell name	Locus names									Figures
			D5S818	D13S317	D7S820	D16S539	VWA	TH01	AM	TPOX	CSF1PO	
	Query (Your Cell)		10,11	11,11	10,11	11,13	14,17	6,8	x,y	8,8	11,12	
1.00(36/36)	CRL-2078	HBE4-E6/E7 [NBE4-E6/E7]	10,11	11,11	10,11	11,13	14,17	6,8	X,Y	8,8	11,12	-
0.78(28/36)	CRL-7240	Hs 325.Ln	11,13	11,11	10,11	11,12	14,17	6,9	X,Y	8,8	10,11	-
0.72(26/36)	CCL-122	HG-261	11,12	9,11	10,11	12,13	16,17	6,9	X,Y	8,8	11,12	-
0.72(26/36)	CRL-2096	CCD-1076Sk	11,11	8,11	10,11	11,13	16,18	6,9.3	X,Y	8,8	11,12	-
0.72(26/36)	CRL-5815	NCI-H727 [H727]	11,12	11,11	8,10	11,13	14,15	8,8	X,X	8,8	11,12	-
0.67(24/36)	242	GAMG	10,11	11,11	9,10	11,13	14,19	6,9.3	X,X	8,8	10,10	-
0.67(24/36)	384	LCLC-103H	11,12	11,11	8,11	13,13	14,16	6,6	X,Y	8,8	10,12	-
0.67(24/36)	497	MEC-1	10,12	11,12	7,11	9,11	16,17	6,8	X,Y	8,11	11,12	-
0.67(24/36)	500	MEC-2	10,12	11,12	7,11	9,11	16,17	6,8	X,Y	8,11	11,12	-

鉴定人员：石勇
鉴定日期：2016-7-25

Cinoasia Institute 上海市邯郸路 100 弄 54 甲 102-104；13916639227
复旦大学科技园；shiyong@cinoasia.com
